# Gender Dimorphism in Aspartame-Induced Impairment of Spatial Cognition and Insulin Sensitivity

**DOI:** 10.1371/journal.pone.0031570

**Published:** 2012-04-03

**Authors:** Kate S. Collison, Nadine J. Makhoul, Marya Z. Zaidi, Soad M. Saleh, Bernard Andres, Angela Inglis, Rana Al-Rabiah, Futwan A. Al-Mohanna

**Affiliations:** 1 Cell Biology and Diabetes Research Unit, Department of Cell Biology, King Faisal Specialist Hospital and Research Centre, Riyadh, Saudi Arabia; 2 Al-Faisal University Medical School, Riyadh, Saudi Arabia; Pennington Biomedical Research Center, United States of America

## Abstract

Previous studies have linked aspartame consumption to impaired retention of learned behavior in rodents. Prenatal exposure to aspartame has also been shown to impair odor-associative learning in guinea pigs; and recently, aspartame-fed hyperlipidemic zebrafish exhibited weight gain, hyperglycemia and acute swimming defects. We therefore investigated the effects of chronic lifetime exposure to aspartame, commencing *in utero*, on changes in blood glucose parameters, spatial learning and memory in C57BL/6J mice. Morris Water Maze (MWM) testing was used to assess learning and memory, and a random-fed insulin tolerance test was performed to assess glucose homeostasis. Pearson correlation analysis was used to investigate the associations between body characteristics and MWM performance outcome variables. At 17 weeks of age, male aspartame-fed mice exhibited weight gain, elevated fasting glucose levels and decreased insulin sensitivity compared to controls (P<0.05). Females were less affected, but had significantly raised fasting glucose levels. During spatial learning trials in the MWM (acquisition training), the escape latencies of male aspartame-fed mice were consistently higher than controls, indicative of learning impairment. Thigmotactic behavior and time spent floating directionless was increased in aspartame mice, who also spent less time searching in the target quadrant of the maze (P<0.05). Spatial learning of female aspartame-fed mice was not significantly different from controls. Reference memory during a probe test was affected in both genders, with the aspartame-fed mice spending significantly less time searching for the former location of the platform. Interestingly, the extent of visceral fat deposition correlated positively with non-spatial search strategies such as floating and thigmotaxis, and negatively with time spent in the target quadrant and swimming across the location of the escape platform. These data suggest that lifetime exposure to aspartame, commencing *in utero,* may affect spatial cognition and glucose homeostasis in C57BL/6J mice, particularly in males.

## Introduction

Previous studies have shown that chronic consumption of the dipeptide artificial sweetener aspartame may affect the T-maze cognitive performance of male rats, promoting impairment of retention of learned behavior when compared to the performance of controls [1]. The Acceptable Daily Intake for aspartame currently stands at 50 mg/Kg body weight in the United States, and 40 mg/Kg in Europe. Once ingested, aspartame (L-aspartyl-phenylalanine methyl ester) is rapidly metabolized to its metabolic components phenylalanine, aspartate, and methanol in the ratio of 50∶40∶10 w/w/w [2]. Whilst the neurological effects of methanol have been well documented, [3] aspartate, like glutamate, has been shown to cause brain lesions [4], obesity [5] and impaired memory retention [6] in rodents exposed to these excitatory amino acids (EAA).

The aspartame metabolite phenylalanine is an essential amino acid which occurs naturally in the breast milk of mammals; however high levels of phenylalanine are a health hazard to those born with phenylketonuria (PKU), a metabolic disorder caused by an inherited mutation in the phenylalanine hydroxylase (PAH) gene which prevents phenylalanine from being metabolized correctly. This results in a detrimental accumulation of the amino acid, leading to developmental defects, seizures and mental retardation [7]. Normal mammalian plasma levels of phenylalanine are approx. 30–50 µM (0.5–0.8 mg/dL), however 1 in 50 individuals are heterozygous for the mutation in the phenylalanine hydroxylase gene [8], resulting in significantly higher levels of fasting plasma phenylalanine compared to non-carriers [9], together with a reduced phenylalanine clearance rate after intravenous loading [10]. Repeated ingestion of 8 servings of aspartame-sweetened beverages by PAH heterozygous individuals incurred plasma phenylalanine levels of up to 165 µM [11], although this was still well below the levels reported to cause neurotoxicity during acute administration in primates. Additionally, genetically mutated PAH-deficient homozygous *Pah* ^enu2^ PKU BTBR mice have six times the level of brain phenylalanine compared to their heterozygous counterparts [12], resulting in abnormal CNS synapses and dendritic spines [13] together with pathological cognitive impairment [14]. *In vitro*, phenylalanine has been demonstrated to specifically and reversibly attenuate glutamatergic synaptic transmission by competing with the glycine binding site of N-methyl D-aspartate (NMDA) receptor [15], [16]. Additionally, the ratio of GluN2A/GluN2B NMDA receptor subunit expression is significantly increased in the hyperphenylalaninemic *Pah* ^enu2^ PKU mouse model, suggesting a potential mechanism whereby elevated levels of phenylalanine may impair brain development and function [12], [17]. Since their discovery in the early 1950s, NMDA receptors have been implicated in many crucial functions of central importance, including learning and memory, neuronal plasticity and neurotoxicity [18], [19]; and they are the only known receptor that is regulated both by a ligand (usually glutamate) and also by voltage [20]. There are at least five binding sites on the NMDA receptor which regulate its activity including glutamate, glycine, magnesium, zinc and a fifth site that binds to the hallucinogenic substance phencyclidine [21]. The central role of NMDA receptors in the process of learning and memory has been confirmed by the extensive use of NMDA receptor agonists and antagonists to study long term potentiation in memory acquisition and maintenance [22].

Whereas it is generally agreed that aspartate crosses the placenta only to a limited degree [23], phenylalanine is actively transported across the placenta [24], resulting in an increase in this aromatic amino acid at the expense of the maternal concentration [25]. During pregnancy, aspartame administration by gavage resulted in impaired performance of the offspring in an odor-aversion test administered to guinea-pigs within the first month of life [26]. This insightful study confirms previous reports in a second species, that aspartame administered pre- and postnatally to rats can result in impaired cognitive performance of the offspring [27]. Furthermore, aspartame has been shown to cause brain inflammation, hyperglycemia and fatalities in a third species: the hyperlipidemic zebrafish model [28]. Aspartame-fed zebrafish also exhibited swimming defects which were interpreted as possibly due to damage in the brain and neurons (28).

The timing, dosage and route of EAA administration *in vivo* appears to be of critical importance, since acute exposure to high doses of dipeptide aspartame during adulthood has no effect on cognitive ability in either humans [29] or rodents [30]. However a single i.p. injection 500 mg/Kg aspartate was sufficient to cause memory impairment and neuronal damage in adult mice undergoing passive avoidance testing [6]; and intracranial injections of phenylalanine caused permanent amnesia in 1 day old chicks [31]. Similarly, perinatal exposure to glutamate results in delayed onset neuroendocrine dysfunction together with cognitive deficiencies [32]–[38], whereas exposure to considerable amounts of dietary glutamate in adulthood is apparently without effect [39]. Interestingly, we [40] and others [41] have noted gender-specific differences in behavior in response to Monosodium Glutamate (MSG). Gender dimorphism in MSG-induced impairment of the growth hormone / IGF-1 axis has also been investigated [42], and it would be of interest to ascertain whether gender dimorphism in glucose homeostasis exists in response to aspartame consumption.

The aims of the present study were therefore to examine the effect of lifetime exposure to aspartame, commencing *in utero*, on weight gain, spatial cognition, insulin sensitivity and glucose parameters of male and female C57BL/6J mice. We used a dosage of aspartame which approximated the ADI for aspartame in the US (approx. 50 mg/kg body weight). Insulin sensitivity was assessed by a random fed insulin tolerance test (ITT), together with measurements of fasting glucose and insulin levels; and cognitive performance was assessed in the Morris Water Maze (MWM). The relationship between visceral fat deposition and cognitive function was determined by analyzing the correlation between body characteristics, glucose and insulin parameters and performance targets in the MWM test, using Pearson correlation analysis.

## Materials and Methods

### Animals and Diets

C57BL/6J mice of both sexes were obtained from the Jackson Laboratory and housed/caged in a controlled environment (3 to a cage in pathogen-free conditions of 12 h light/dark cycle, 22±2°C) and fed a standard chow diet with water *ad libitum* until 6 weeks of age. The two diet groups used in this study were (1) *ad libitum* Standard Chow (Control diet) with *ad libitum* drinking water. (2) *Ad libitum* Standard Chow, with *ad libitum* drinking water containing 0.25 g/L aspartame as the only source of drinking water (Asp-Phe methyl ester, catalog A5139 Sigma Aldrich). After a 3-week period of adjustment, male and female mice were bred, weaned and maintained on these respective diets for the times stated. Mean body weight was assessed at 6 and 17 weeks of age. Food and water intake was assessed at 7 weeks and again at 15 weeks of age, in pre-weighed mice over a four day period, by weighing the food pellets and water bottles to the nearest 0.1 g. Mean food/water consumption was calculated by subtraction, and expressed as g/20 g body weight (bw) and mls/20 g bw respectively. Mean aspartame consumption was calculated from the amount of aspartame-water consumed, and expressed in mg per Kg bw. Body length to the nearest mm was assessed at 6 and 17 weeks of age using a woven tape measure. The breeding and care of the animals were in accordance with the protocols approved by the Animal Care and Use Committee of the King Faisal Specialist Hospital & Research Centre. At the conclusion of the study, animals were humanely euthanized with a mixture of xylazine and ketamine (10 mg/kg and 100 mg/kg respectively); and the liver and visceral fat were carefully dissected out and removed, rinsed twice in PBS, blotted dry and weighted to the nearest 0.01 g. These tissues were rapidly snap-frozen for use in further studies.

### Measurement of Fasting Serum Glucose, Insulin and Lipid Profile

Overnight fasting blood glucose was measured using the Ascensia Contour glucometer (Bayer HealthCare, IN, USA). Fasting serum insulin was measured using the mouse insulin ELISA kit from Millipore/Linco (Uppsala, Sweden). Homeostatic Model Assessment Index (HOMA-IR) values, a measure of insulin resistance, were calculated according to the established formula: (fasting serum insulin µIU/ml) * (fasting serum glucose mM)/22.5 [43]. Serum Triglyceride (Tg), total cholesterol (T-CHOL), and HDL-C concentrations were measured in overnight fasted mice using the Reflovet Plus instrument (Roche, F. Hoffmann-La Roche Ltd, Basel, Switzerland) as previously described [40].

### Morris Water Maze (MWM) Testing Apparatus

The spatial learning abilities of the C57BL/6J mice were assessed at 16 weeks of age (mature adulthood) in a MWM task [44], since our previous studies indicate that this is the optimal time for assessing the effect of dietary interventions on cognitive behavior [40]. The apparatus consisted of a white circular pool of 150 cm diameter and 50 cm height, filled with water made opaque by the addition of a small amount of non-toxic white paint (30 cm deep) and maintained at 21–22°C. A circular escape platform (11 cm in diameter) was placed in a fixed South-West location hidden 0.5 cm below the surface of the water, and 3 stationary geometric visual cues were kept in the room throughout the period of testing as previously described [40]. A digital camera was positioned above the centre of the tank and linked to a tracking system in order to record the performance of the experimental subjects (HVS Image Analysis VP-200, HVS Image, Hampton, UK).

### MWM Procedure: Assessment of Learning abilities

Mice were given four consecutive days of acquisition training sessions that consisted of four trials per day with an inter-trial interval of 20 minutes. In order to investigate the effect of diet on allocentric spatial reference memory, the position of the hidden platform remained fixed however the entry point was pseudo-randomly selected from one of the 4 compass locations each day, and the same sequence of starting points was used for all the mice tested. Mice were given 120 seconds to find the platform and if the subject failed to locate the platform within this period, it was guided onto it. All mice were allowed to rest on the platform for a 30 second interval after each trial. At the end of the training block, mice were put in a pre-warmed drying cage and allowed to dry prior to being returned to their experimental cages.

### MWM Escape Strategies Analysis

During the acquisition phases of learning, mice adopt a number of increasingly sophisticated escape strategies as part of allocentric spatial memory acquisition [45]. Initial strategies such as thigmotaxis and random searching which do not involve any spatial or directional preference progress to strategies usually described as scanning (directionless searching of the interior portion of the pool), chaining (circular swimming at an approximately fixed distance greater than 16 cm from the wall), followed by directed searching for the escape platform.

### MWM Probe Test for Spatial Memory Assessment

A Probe Test was run on day 5, after 4 days of acquisition. Before the probe test, the hidden platform was removed and the mouse was introduced from the north quadrant, where it was left to search for the platform for 60 seconds. Time spent in the target quadrant, number of platform crossings, and annulus crossing index (ACI: defined as the number of crosses over the platform position in the target quadrant, relative to crosses over the corresponding platforms in the remaining three quadrants) were recorded.

### Random-fed Insulin Tolerance Test (ITT)

The effect of aspartame on glucose parameters was determined using a random-fed insulin tolerance test (ITT) administered to 19-week old mice. Because swimming exercise has been shown to alter insulin sensitivity and glucose homeostasis [46], we performed the ITT on additional mice who had not been subjected to the water maze protocol (n = 18 per diet / gender group). For the ITT, an intraperitoneal injection of insulin (Sigma, IL) at a dose of 0.75 U/kg body weight was administered, and whole blood glucose levels were measured from the tail vein at 0, 15, 30, 45 and 60 minutes after injection.

### Data Analysis

Data were presented as mean ±SEM for body-weight, serum lipid profile, glucose and insulin variables separately in male and female mice. For statistical comparisons between the two diet groups, unpaired Student’s t test was applied using Graph Pad Instat software (version 3, California, USA). For the MWM experiments, the following variables characterizing the performance of mice in the MWM were chosen for analysis: Latency, defined as time taken (in seconds) for a mouse to reach and climb the platform, and the length of each Swim Path (in meters). Locomotor activity was analyzed using the average Swim Speed (M/s), and the percent of time that the animals were relatively stationary (with swim speed below the 0.06-m/sec threshold) was recorded as Floating Time (%). Thigmotaxic swimming activity was defined as percent of time swimming parallel to the pool’s wall within a 16-cm distance from the wall. Spatial cognition of the platform location during acquisition trials and during the probe day was evaluated by the analysis of the dwelling time in each of the pool’s quadrants. Overall treatment effects were examined using a repeated measures generalized linear model using SPSS 13.0 statistical software (SPSS Inc., Chicago, IL). For the analysis of the escape strategies, the Swim Paths for each mouse was plotted and categorized into one of the following search strategies:(1) Direct swim towards the location of the platform; (2) Directed search, in which the animal swims in a search pathway directly towards the platform; (3) Focal search: searching in the quadrant containing the hidden platform. (4) Scanning, where the search path is restricted to a limited, often central, area of the pool; (5) Random searching in which the animal swims over the entire area of the pool. (6) Chaining, which is circular swimming at a fixed distance from the wall; (7) Thigmotaxis (wall-hugging swim): a persistent swim along the outer 16 cm of the pool. (8) Floating: a state of inactivity without forward movement. The use of each search strategy was presented as a percent of incidences during each trial over the whole analyzed experimental period.

## Results

### Effect of Aspartame on Weight Gain, Blood Glucose and Insulin Parameters

Averaged aspartame ingestion in the drinking water was 55.14±4.74 mg/Kg body weight. Water and food consumption were unaffected by the addition of aspartame compared to controls (data not shown). Weight gain was significantly higher in the male aspartame diet group compared to controls (Table 1, P = 0.024), but female weight gain was unaffected by diet. Body length and visceral fat deposition were both increased in aspartame-fed mice of both sexes (Table 1, P<0.01). Females weighed less than males, and had less fat deposition following aspartame treatment, although there was no difference between the absolute or relative weight of the visceral fat collected from control animals. Liver weight was affected by diet as well as gender, with livers from aspartame-fed males weighing more than control males; and more than those of female aspartame-fed mice (P<0.05 and P<0.01 respectively). Additionally, aspartame-fed mice had elevated fasting blood glucose levels compared to non-consumers of both sexes, although females had lower levels than males (Table 1, P<0.01). Fasting insulin and HOMA-IR levels in female mice were significantly lower than males, although all were within the normal range (Table 1). Pearson correlation analysis was used in order to examine associations between body weight, fat deposition, indices of glucose homeostasis and lipid profile. As expected, body weight at six weeks of age strongly correlated with weight at 17 weeks (Table 2, r = 0.888, P<0.001). We also found correlations between weight gain and visceral fat, body length, fasting glucose, insulin and HOMA-IR (Table 2, r = 0.322, 0.546, 0.440, 0.371 and 0.548 respectively, P<0.01).

**Table 1:** Effect of aspartame consumption on weight gain, adiposity, glucose homeostasis and lipid profile.

	Male						Female					
	*Control*			*Aspartame*			*Control*			*Aspartame*		
Body Weight (6 weeks)	17.6	±	0.13	17.22	±	0.35	14.31^§§^	±	0.17	14.34^§§^	±	0.22
Body Weight (17 weeks)	24.02	±	0.48	24.96	±	0.4	18.91^§§^	±	0.21	19.42^§§^	±	0.27
% Weight Gain	36.42	±	2.44	45.31*	±	2.71	32.3	±	1.45	35.63^§§^	±	1.5
Length (cm)	9.65	±	0.08	10.04**	±	0.08	9.21	±	0.07	9.52**^§§^	±	0.06
Visceral Fat (g)	0.09	±	0.02	0.24**	±	0.02	0.08	±	0.01	0.13**^§§^	±	0.01
RW of Visceral Fat (g/20g BW)	0.08	±	0.01	0.192**	±	0.02	0.09	±	0.01	0.13**^§§^	±	0.01
Liver Weight (g)	0.89	±	0.02	1.03*	±	0.06	0.76	±	0.03	0.71^§§^	±	0.02
RW of Liver (g/20g BW)	0.75	±	0.02	0.82	±	0.04	0.81	±	0.03	0.736*	±	0.02
Fasting Glucose (mM)	3.1	±	0.18	4.61**	±	0.43	2.93	±	0.13	3.65**^§^	±	0.17
Fasting Insulin (uIU/ml)	17.32	±	2.54	16.67	±	1.86	10.37^§^	±	0.8	8*^§§^	±	0.58
HOMA-IR	2.36	±	0.35	3.19	±	0.24	1.35^§^	±	0.11	1.29^§§^	±	0.1
TG (mg/dL)	104.17	±	7.42	107.41	±	5.88	123.25	±	6.67	98.24*	±	6.32
T-CHOL (mg/dL)	121.5	±	1.08	121.08	±	1.4	121.92	±	0.91	122	±	0.46
HDL-C (mg/dL)	77.13	±	3.55	63.25**	±	3.04	54.47^§§^	±	2.73	41.45**^§§^	±	2.23
LDL (mg/dL)	23.54	±	3.10	36.34*	±	3.42	42.80	±	2.63	60.89**^§§^	±	2.41

BW, Body Weight; RW, Relative weight.

Data presented are means ± SEM, n = 12 per group. P-value <.05 and < 0.01 based on t-test comparisons of diet groups within sexes are indicated by * and **; and comparison of sexes by § and §§ respectively.

**Table 2:** Correlations between body weight, visceral fat, parameters of glucose homeostasis and lipid profile.

	Body Weight (6 wks)	Body Weight (17 wks)	Weight Gain (%)	Visceral Fat (g)	Length (cm)	Liver Weight (g)	Glucose (mM)	Insulin (uIU/ml)	HOMA-IR	HDL-C (mg/dL)	LDL (mg/dL)	T-CHOL (mg/dL)
Body Weight (6 wks)	1											
Body Weight (17 wks)	0.888***	1										
% weight gain	0.172	0.605***	1									
Visceral Fat (g)	0.372**	0.446**	0.322*	1								
Length (17 wks)	0.649***	0.778***	0.546***	0.567***	1							
Liver Weight (g)	0.619***	0.670***	0.363*	0.423**	0.547***	1						
Glucose (mM)	0.203	0.370*	0.440**	0.403**	0.485***	0.484***	1					
Insulin (uIU/ml)	0.584***	0.654***	0.371**	0.042	0.449**	0.248	−0.095	1				
HOMA-IR	0.638***	0.796***	0.584***	0.288*	0.650***	0.493***	0.468**	0.821***	1			
HDL-C (mg/dL)	0.625***	0.612***	0.236	−0.003	0.215	0.513***	0.121	0.274	0.363*	1		
LDL (mg/dL)	-0.649***	-0.590***	−0.144	0.026	−0.240	−0.478**	−0.024	−0.336*	−0.365*	−0.926***	1	
T-CHOL (mg/dL)	−0.168	−0.093	0.104	−0.058	−0.257	0.040	0.139	−0.300*	−0.157	0.233	0.025	1
TG (mg/dL)	−0.015	−0.109	−0.216	−0.131	−0.120	−0.046	−0.209	−0.021	−0.100	0.020	−0.311*	−0.021

Significant correlations are shown in bold with *, ** and *** indicating a P-value of <.05, <.01 and <.001 respectively, n = 12 per group.

**Figure 1:**
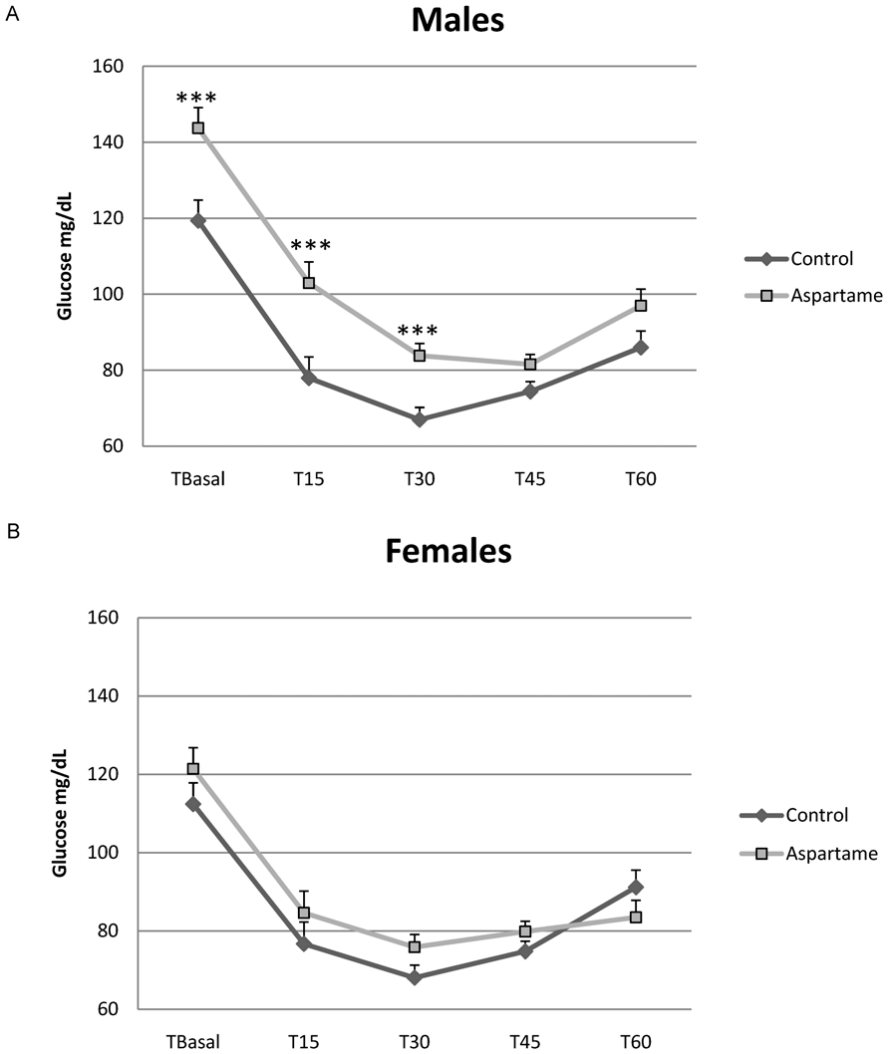
Aspartame consumption reduces insulin sensitivity of male C57BL/6J mice during a random-fed insulin tolerance test. Values are mean±SEM of (A) male glucose levels and (B) female glucose levels, n = 18 per group, *** P<0.001 compared to controls.

A Random-fed insulin tolerance test administered at 19 weeks of age showed that glucose levels in male aspartame-fed mice were 120.2% higher than control mice following insulin challenge (Fig 1A, P = 0.001); and remained significantly elevated above controls for up to 30 minutes, suggesting impairment of glucose and insulin regulation. The mean Area Under the Curve (AUC) in male aspartame-fed mice was significantly higher than control, suggesting deregulation of glucose homeostasis (Table 3: 5829.79±197.72 µU/L/60 min vs 4829.58±131.96 µU/L/60 min: P<0.001), Mean AUC in female mice showed a similar trend without reaching significance (5194.63±208.04 µU/L/60 min vs 4819.93±190.36 µU/L/60 min). The half-life of glucose in the aspartame mice showed a non-significant trend towards elevation compared to control (Table 3). Collectively these data indicate that aspartame treatment affects C57BL/6J weight gain (6–17 weeks of age), visceral fat deposition and glucose homeostasis particularly in males, and to a lesser extent in females.

**Table 3:** Effect of aspartame consumption on blood glucose levels during a random–fed insulin tolerance test.

	Control						Aspartame				P-value	
*Males*												
AUC_total_ (mM x min)	4829.58	±	131.96			5829.79		±	197.07	<.001		
K_glucose_ (%/min)	2.92	±	0.28			2.32		±	0.29	0.15		
T½ (min)	39.69	±	14.05			51.61		±	16.63	0.59		
*Females*												
AUC_total_ (mM x min)	4819.93	±	190.36			5194.63		±	208.04	0.19		
K_glucose_ (%/min)	2.71	±	0.37			2.24		±	0.26	0.31		
T½ (min)	34.19	±	4.4			23.28		±	9.59	0.31		

AUC, area under the curve; K, clearance rate; T½, half-life.

Values are means±SEMs, n = 18 per group.

### Gender Dimorphism in Aspartame-Induced Impairment of Learning

#### Spatial learning strategies in the MWM

**Figure 2:**
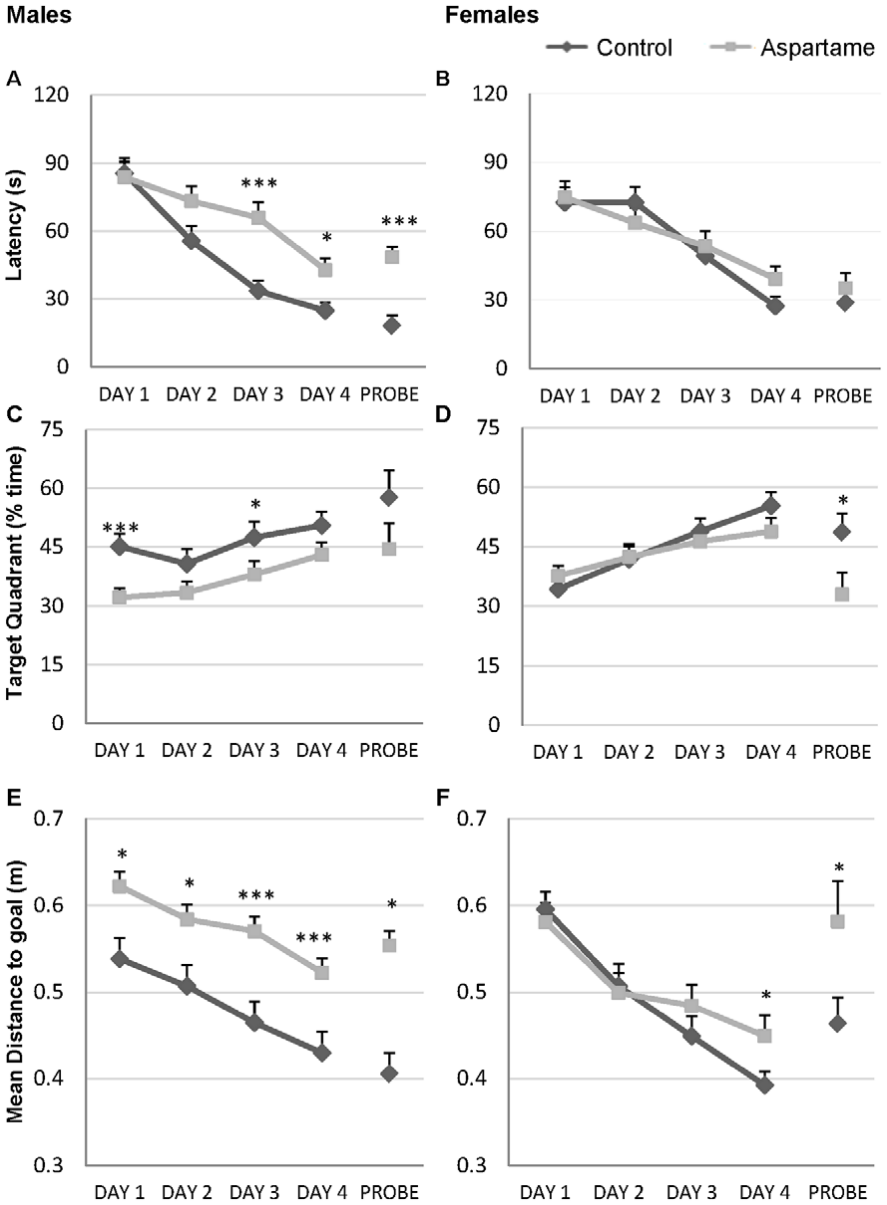
Effect of aspartame consumption on spatial learning in C57BL/6J mice: gender-specific differences. Acquisition curves of escape latency in male (A) and female (B) aspartame-fed and control diet mice. Percentage time spent in target quad in male (C) and female (D) mice. Mean Distance to goal in male (E) and female (F) mice. Each group consisted of 12 mice; * P<0.05, ** P<0.01, *** P<0.001.

During the acquisition phase of the MWM testing (days 1 to 4), significant differences in place learning performance of aspartame-fed mice became apparent. Cued escape latencies across sessions of mice from both diet groups are shown in Fig 2. All mice improved their performance over the 4 days of training, however, latencies of male aspartame-treated mice were higher than control latencies on the last 2 days of the 4-day trial (Fig 2A, P<0.05), suggesting that aspartame may have impaired learning (acquisition) skills in these mice. Female aspartame-fed mice had similar latencies to non-consumers (Fig 2B). The duration of time spent in the target quadrant where the hidden platform was located is shown in figure 2C (males) and 2D (females). As testing progressed, mice learned to spend more time searching for the platform within the target quadrant, but aspartame-fed males spent significantly less time searching for the platform in the target quadrant (Fig 2C, P<0.05). A comparison of mean distance (in meters) to reach the goal platform showed that aspartame-fed mice were farther from their goal than controls on all four days of acquisition training, suggestive of impaired learning (Fig 2E, P<0.05). Swim speed was unaffected by diet (data not shown).

#### Floating and thigmotaxis behavior

**Figure 3:**
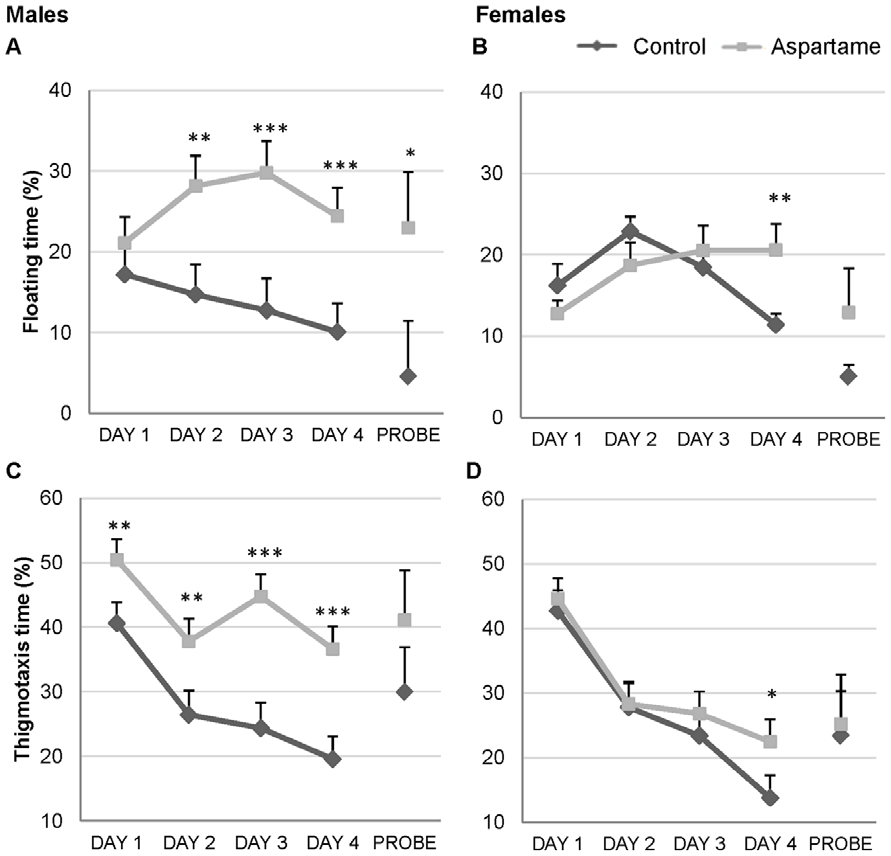
Effects of aspartame consumption on non-cognitive behavior. Percentage of time spent floating in male (A) and female (B) aspartame-fed and control diet mice. Thigmotaxis in male (C) and female (D) mice, n = 12 per group; * P<0.05, ** P<0.01, *** P<0.001.

To gain further insight into the reduced spatial learning of aspartame-fed mice, we studied the non-spatial behavioral parameters of these mice and that of controls. Floating behavior, characterized by periods of immobility and defined as percent of time with swim speed below a 0.06m/sec threshold, revealed a significant effect of diet during the acquisition period, with male aspartame-fed mice exhibiting increased floating behavior on days 2,3 and 4 compared to controls (Fig 3A, P<0.001). Thigmotaxis, defined as percentage of time spent swimming within 16cm of the pool wall, was greatly increased in aspartame-treated males throughout the whole experiment (Fig 3C, P<0.01), whereas female thigmotactic behavior decreased throughout the acquisition period, and showed no diet effect. Taken together, our data suggests that males exposed to chronic dietary aspartame had impaired spatial learning abilities together with increased non-spatial strategies.

#### Effects of aspartame on qualitative aspects of learning

**Figure 4:**
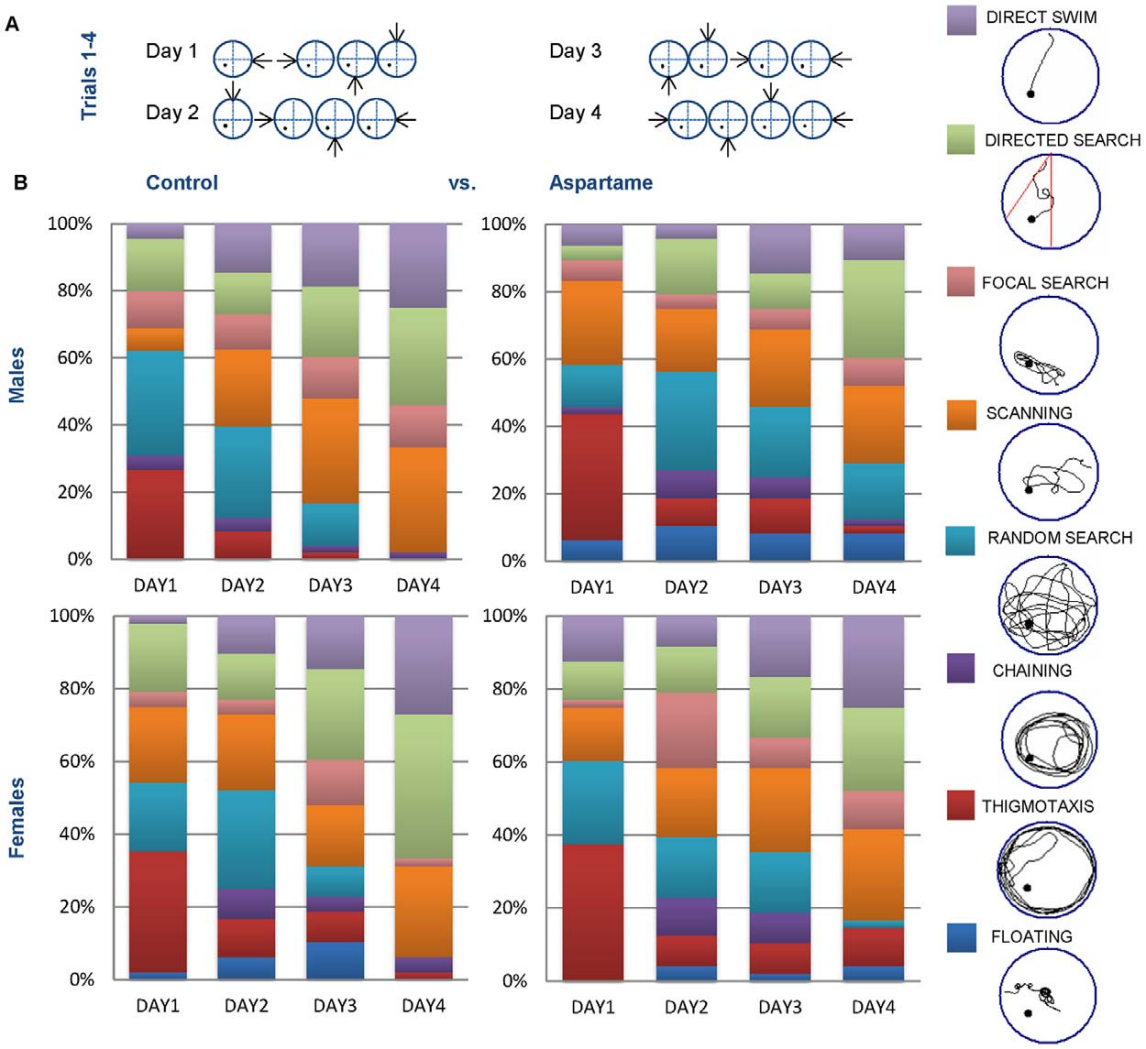
Effects of diet and gender on spatial and non-spatial escape strategies during the MWM test. (A) Orientation of entry point and escape platform on trial days 1–4. Arrows indicate location of entry point. (B) Distribution of search strategies in aspartame-fed mice compared to controls on trial days 1–4. As the acquisition training advanced, control C57BL/6J mice exhibited progressive behavioral changes from random chaining, thigmotaxis and floating into predominantly spatial strategies such as direct swim and direct search. Mice in the aspartame group exhibited less spatial strategies and more non-spatial behavior throughout the trials.

To assess the effect of diet on qualitative aspects of learning which could account for the apparent differences in latency, we analyzed the respective search strategies displayed by the mice to locate the hidden platform on the four days of acquisition training. As the training progressed, control male and female mice spent increasing amounts of time in directed searching and direct swimming towards the platform, in addition to using other strategies which require spatial and directional searching, including focal searching (Fig 4A,B). Non-spatial behavior, such as thigmotaxis and floating, decreased during the 4 days of training such that by Day 4, male control mice spent no time either swimming around the periphery of the maze, or floating directionless. Conversely, compared to control, aspartame-fed mice exhibited significantly less time acquiring spatial and directional strategies to find the hidden platform during the acquisition training (Fig 4B, P<0.05).

#### Effect of aspartame on spatial memory in the MWM Probe Test

**Figure 5:**
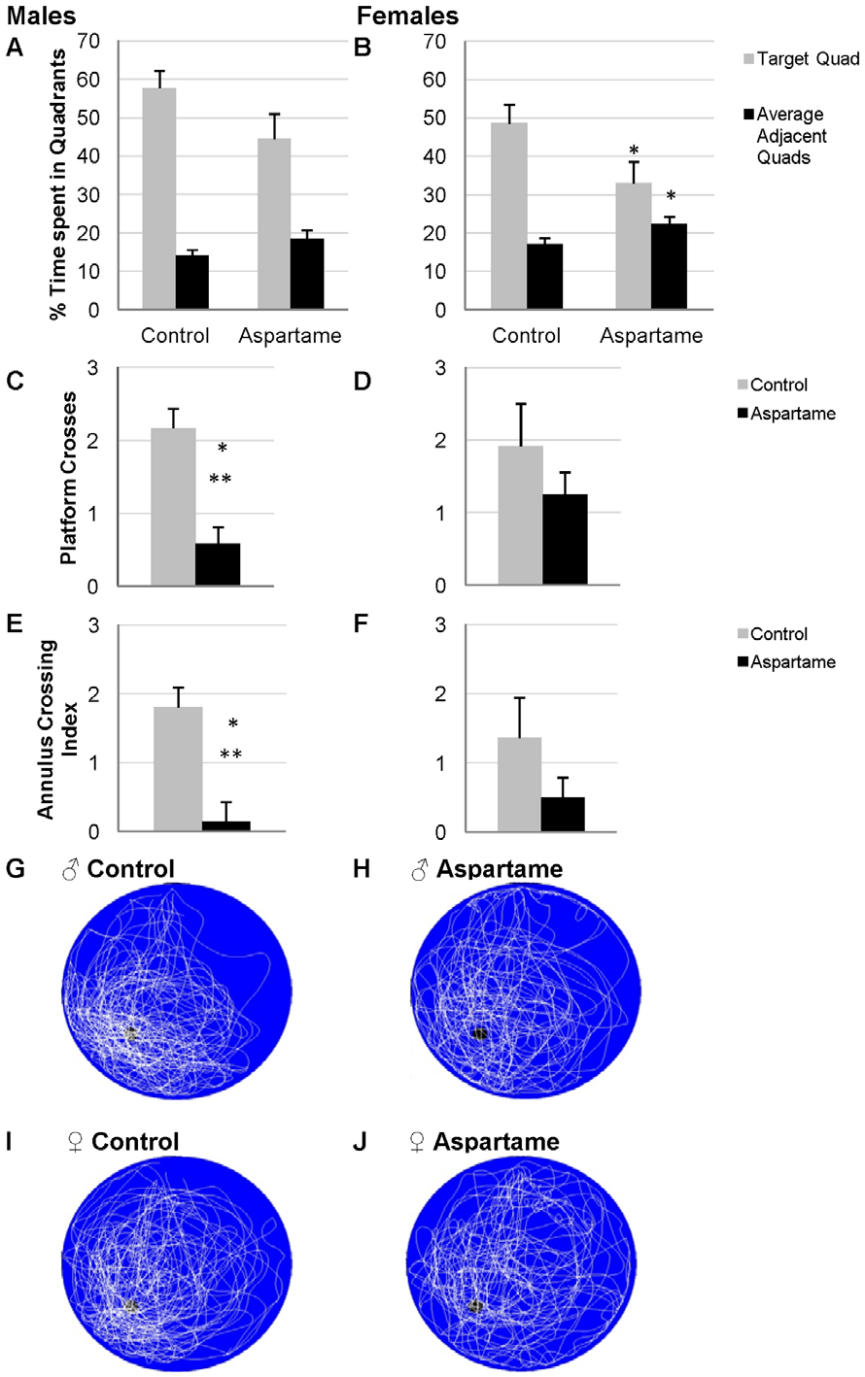
Results from a probe trial intended to measure spatial memory. Percentage of time spent searching in the former location of the platform (Target Quad) in (A) males and (B) females, compared to time spent in the adjacent quads. Aspartame-fed males showed a reduction in the number of times they crossed over former location of the platform (C: Platform Crossings and (E: Annulus Crossing Index), n = 12 per group, * P<0.05, ** P<0.01, *** P<0.001. There was no significance in the reduction of platform crossings and Annulus Crossing Index in females. Actual overlapping swim paths of the control (G,I) and aspartame-fed mice (H,J). Swim paths illustrate less intense swimming in the location of the platform by aspartame-fed mice, compared to controls.

Regardless of gender, diet-induced differences in spatial retention were apparent during a probe test, the recognized measure of spatial memory, performed after the 4 days of acquisition training. Once the platform had been removed, aspartame-fed mice of both genders spent significantly less time swimming towards the former position of the hidden platform compared to control, (Fig 5A & B, P<0.05). Additionally, male aspartame-fed mice crossed the former location of the platform significantly less frequently than controls (Fig 5 C, P<0.01), and had a lower Annulus Crossing Index (Fig 5E, P<0.01), implying impairment of spatial memory. Platform crossing and annulus crossing indices of female mice showed a similar trend without reaching statistical significance (Fig 5 D & F). Overlapping swim paths (n = 12) of aspartame-fed and control male and female mice are shown in figure 5 G & H. The location of the hidden platform can be visualized through the aspartame-mice swim path, indicating that the animal did not frequently cross the location of the platform during the trial.

To complete our analysis of the performance of the mice during this water maze test, we next used repeated measures generalized linear modeling analysis of MWM performance variables throughout the 4 days of acquisition training and during the probe test, in males and females consuming the aspartame diet compared to controls (Table 4). In males, significant differences in floating and thigmotactic behavior between controls and aspartame-fed mice were apparent on four out of the five days of testing, and the mean distance to goal (a measure of how close mice swam towards the platform) was significantly different between the two diet groups on all days of testing (P<0.05). The performance of females however, was only significantly different on the last day of acquisition training and during the probe test.

**Table 4:** Generalized linear modeling of performance variables in aspartame-fed C57BL/6J mice compared to controls.

		Day 1		Day 2		Day 3		Day 4		Probe Test	
Males											
Latency		0.862		0.061		**<.001**		**<.01**		**<.001**	
Thigmotaxis time (%)		**<.01**		**<.01**		**<.0001**		**<.0001**		0.236	
Floating time (%)		0.348		**<.01**		**<.001**		**<.001**		**<.05**	
Mean distance to goal (m)		**<.05**		**<.05**		**<.001**		**<.001**		**<.05**	
Time in target quadrant (%)		**<.01**		0.076		**<.05**		0.112		0.112	
Time in non-target quadrants (%)		**<.01**		0.076		**<.05**		0.112		0.111	
Platform Crosses		0.715		0.547		**<.001**		0.800		**<.001**	
Annulus Crossing Index		0.781		0.569		**<.01**		0.553		**<.001**	
Females											
Latency		0.817		0.359		0.644		0.090		0.474	
Thigmotaxis (% time )		0.623		0.957		0.452		**<.05**		0.818	
Floating (% time)		0.191		0.347		0.640		**<.01**		0.175	
Mean distance to goal (m)		0.626		0.822		0.291		**<.05**		**<.05**	
Time in target quadrant (%)		0.321		0.897		0.605		0.176		**<.05**	
Time in non-target quadrants (%)		0.321		0.895		0.602		0.175		**<.05**	
Platform Crosses		0.225		0.339		0.650		0.153		0.277	
Annulus Crossing Index		0.263		0.536		0.290		0.619		0.199	

Significant differences in performance between the control and the diet groups are indicated in bold P-value ≤ 0.05, for trial days 1 to 4 and probe day, n = 12 per group.

### Correlations between Water Maze Performance Output Variables and Body Characteristics

In order to understand more about the possible mechanisms behind the reduction in escape latencies in aspartame-fed mice, we next examined correlations between body characteristics and MWM performance indicators (Table 5). Surprisingly visceral fat deposition correlated positively with floating time on all days of the trials, and also with thigmotaxis on day 3,4 and the day of the probe test (P<0.05). Fat deposition also correlated with escape latency time on these days, and negatively with time spent in the target quarter, platform crossings and ACI. Finally, in order to validate our analysis, we performed correlation analysis on all MWM outcome variables in all mice analyzed simultaneously. As expected, we found a strong negative correlation between latency and floating time and between latency and mean distance to goal (Table S1). Escape latency also correlated negatively with time in target quadrant, number platform crossings, and the ACI. Taken together, these correlations suggest that our analysis is valid, and lends credence to observations concerning adiposity and non-spatial learning strategies.

**Table 5:** Summary of correlation analysis between body characteristics and spatial memory variables in the MWM test.

		% weight gain		Body Weight (g)		Visceral Fat (g)		Insulin (uIU/ml)	Glucose (mM)	HOMA-IR	T-CHOL (mg/dL)	HDL-C (mg/dL)	LDL (mg/dL)
Latency (s)													
Day 2			0.027		−0.092	0.005	0.095		0.050	0.132	0.242	−0.054	0.094
Day 3			0.232		0.075	0.357*	0.023		0.246	0.148	0.191	−0.065	0.130
Day 4			0.063		0.029	0.428**	−0.076		0.124	0.010	0.195	−0.070	0.139
	Probe Test		0.211		0.076	0.431**	0.017		0.207	0.150	−0.116	−0.257	0.281*
Thigmotaxis time (%)													
Day 2			0.290*		0.113	0.265	0.085		0.187	0.195	0.097	0.053	−0.025
Day 3			0.327*		0.263	0.488***	0.002		0.409**	0.255	0.206	0.169	−0.108
Day 4			0.163		0.291*	0.553***	0.046		0.396**	0.240	0.048	0.094	−0.020
Probe Test			−0.010		0.211	0.356*	0.001		0.259	0.133	0.061	0.123	−0.097
Floating Time (%)													
Day 1			−0.157		0.046	0.402**	−0.115		−0.045	−0.089	0.096	0.088	−0.058
Day 2			−0.103		−0.052	0.340*	−0.134		0.174	0.005	0.111	−0.074	0.104
Day 3			0.086		0.062	0.487***	−0.224		0.369**	0.006	0.278*	−0.045	0.158
Day 4			0.162		0.054	0.441**	−0.128		0.349*	0.076	0.214	−0.035	0.156
Probe Test			0.258		0.188	0.457**	−0.077		0.433**	0.177	0.202	0.012	0.074
Time in Target Quadrants (%)													
Day 1			−0.164		0.075	−0.284*	0.136		−0.234	−0.024	−0.164	0.158	−0.230
Day 2			−0.245		−0.224	−0.188	−0.361*		−0.078	−0.355*	−0.188	−0.102	0.086
Day 3			−0.137		−0.162	−0.349*	−0.135		−0.021	−0.121	−0.056	0.037	−0.041
Day 4			−0.085		−0.243	−0.294*	−0.193		−0.302*	−0.330*	0.167	0.024	0.048
Probe Test			−0.054		0.160	−0.134	−0.057		−0.002	−0.055	−0.114	0.347*	-0.368**
Platform Crosses													
Day 2			−0.028		0.130	0.071	−0.020		−0.010	−0.034	−0.387**	0.034	−0.122
Day 4			−0.122		0.002	−0.369**	0.094		−0.260	−0.051	−0.160	0.079	−0.146
Probe Test			−0.265		−0.120	−0.401**	0.023		−0.157	−0.080	−0.005	0.223	−0.221
ACI													
Day 3			−0.188		−0.118	−0.384**	−0.051		−0.182	−0.131	−0.030	0.042	−0.073
Day 4			−0.133		−0.199	−0.275*	−0.173		0.060	−0.125	−0.161	−0.118	0.079
Probe Test			−0.213		−0.027	−0.359*	−0.004		−0.074	−0.052	0.048	0.317*	−0.303*

Significant correlations are shown in bold with *, ** and *** indicating a P-value of ≤.05, ≤.01 and ≤.001 respectively, n = 12 per group

## Discussion

Our results suggest that neonatal exposure to aspartame consumed as part of the diet of pregnant mice, together with continued chronic exposure of the offspring to dietary aspartame throughout the first 20 weeks of life, may result in increased weight gain compared to controls together with impairment of insulin sensitivity and cognitive performance, most notably in males. Food and water intake was not affected by aspartame administration within the ADI. The results of our analysis support previous observations that aspartame may cause impairment in learning and memory particularly when administered chronically [1], [47] or neonatally [26]. Additionally, damage to hypothalamic morphology has been reported in neonatal rodents ingesting high amounts of aspartame [48].

Aspartame is metabolized rapidly into methanol, phenylalanine and aspartate [2]; and oral administration of aspartame (200 mg/Kg) has been shown to increase levels of rat brain phenylalanine and its metabolite tyrosine, whilst decreasing levels of leucine, isoleucine and valine [49]. Whereas it is generally accepted that aspartate does not readily cross the placenta, phenylalanine and tryosine are readily transported to the fetal tissues; resulting in an increase in phenylalanine at the expense of the maternal concentration [25]. In rodents, phenylalanine is readily converted into the neurotransmitter precursor tyrosine by the hepatic enzyme PAH [50]; however if the activity of this enzyme is reduced or absent, the high levels of accumulated phenylalanine may be converted into other metabolites such as phenylpyruvate, phenylacetate and phenyllactate [50], [51]. Crucially, studies have shown that in rodents, PAH activity is undetectable until the final days of gestation and birth, whereupon the gene is activated by glucocorticoids and cyclic AMP [52]–[54]. Experimentally induced hyperphenylalaninemia has been shown to result in spatial and non-spatial deficits in cognition and learning that are not related to impairment of locomotor skills [55], [56]. In humans, deficiency of PAH due to genetic mutations in the gene results in phenylketonuria (PKU), which is characterized by neurotoxic hyperphenylalaninemia and microcephaly, together with visuo-spatial, executive and attention deficits [57], [58]. The mechanisms responsible for the hyperphenylalaninemia-induced brain damage are still largely unknown; however hyperphenylalaninemia has recently been shown to promote oxidative stress in rodent brains, which may contribute to the neurotoxicity in phenylketonuria [59], [60]. Oxidative stress is the result of the aberrant production of reactive oxygen and / or nitrogen species, or a decrease in the capacity of antioxidant defenses for example glutathione; and has been linked to a number of neurodegenerative diseases and to the cognitive decline associated with aging [61]. Importantly, subcutaneous injections of aspartame have recently been shown to increase rat brain thiobarbituric acid-reactive substances (TBARS; markers of lipid peroxidation) and decrease glutathione levels [62]. Collectively, these observations may provide clues as to a mechanism whereby aspartame metabolites; phenylalanine in particular, may contribute to the impairment in spatial learning and memory that we observed in mice exposed to aspartame *in utero* and during the first months of life.

During acquisition of the water maze task, young rodents typically improve their performance as indicated by a progressive reduction in escape latencies over successive training sessions. Upon introduction to the maze, mice initially adopt non-spatial behaviors including thigmotaxis, scanning and chaining [45], [63]. As training progresses, this behavior gives way to spatial learning, resulting in more cued swimming towards the hidden platform, more time spent in the target quadrant, and shorter escape latencies. Within the context of this paradigm, aspartame-fed mice exhibited significant differences in learning strategies at four months of age, which resulted in longer escape latencies compared to controls, together with quantitative and qualitative differences in behavioral strategies employed. Towards the end of acquisition training, aspartame-fed mice spent significantly more time swimming around the periphery of the pool and passively floating compared to control mice, which may be indicative of an ineffectual non-spatial swim strategy [64]. Increased thigmotaxis behavior linked to a deficit in responding to visual cues has previously been noted after experimentally induced lesions to the dorsal-striatum: a compound structure of the brain believed to be involved in stimulus-response learning [65]. Additionally, lesions to the hippocampus [66] and NMDA receptor blockade using specific antagonists have also shown to result in increased thigmotactic behavior [67], [68].

Impairment of spatial memory in the aspartame diet group was suggested by a significant reduction in time spent swimming towards the former platform location during the probe test. Additionally, thigmotactic behavior and passive floating during the probe test was increased in male aspartame-fed mice compared to controls. Taken together this suggests that chronic exposure to aspartame may impair rodent spatial memory. It has previously been suggested that exposure to high doses of aspartame at a late stage of pregnancy may result in a delay in visual placing response in the offspring, which is a measure of sensorimotor activity [69]. However, a second study failed to duplicate these findings [70].

In addition to the effects of aspartame on rodent cognitive performance in the water maze, aspartame appeared to raise fasting blood glucose levels in both sexes. The relationship between peripheral blood glucose levels and cognition has been well-documented and suggests that there is a homeostatic neuroglycemic range within which optimal cognitive function occurs [71]. Hyperglycemia may damage the microvasculature of the blood-brain barrier and/or modify insulin availability in the brain, disrupting normal brain function and cognition. Interestingly hyperglycemia, increased body weight and swimming defects were recently observed in hyperlipidic zebrafish exposed to aspartame [28], which tends to support our present observations. However, our data using doses of aspartame approximating the current ADI contrasts with a previous report which concluded that long term consumption of aspartame from six weeks onwards did not increase weight gain [72]. This apparent contradiction could conceivably be due to the fact the aspartame in that toxicology study was administered from the 6^th^ week of life onwards at a dose of 2–4 g/Kg body weight (40–80 times the current ADI), resulting in a significant reduction in food intake for animals consuming the higher quantity of the dipeptide sweetener. Interestingly human studies have found a positive correlation between the consumption of artificial sweeteners and weight gain [73], [74]; and surprisingly in diabetic subjects, aspartame-containing meals elevated blood glucose and insulin levels to the same extent as that of higher-calorie sucrose-containing meals [75].

A third novel observation in our study relates to gender-specific differences in insulin sensitivity and cognitive performance, with males apparently exhibiting a greater adiposity, weight gain, glucose dysregulation and cognitive impairment compared to females. Gender-specific differences in behavior have also been documented in response to Monosodium Glutamate (MSG) a commonly consumed food additive. MSG-treated males appear to be more adversely affected than females [41], [76], and have a greater increase in adipose tissue deposition [77] and insulin resistance [34] than females. Therefore the possibility exists that although both males and females showed equal increases in fasting glucose levels in response to aspartame, the gender-specific differences in cognitive performance may be due to differences in the extent of adiposity and insulin resistance, both of which are associated with cognitive performance [78]. A final intriguing outcome from our study was the correlation we found between visceral adiposity and the adoption of non-spatial escape strategies (thigmotaxis and floating behavior) during the MWM test. In general, non-spatial behavior in the MWM test is associated with an inability to adopt spatial cognitive abilities [79], and others have found that high-fat diets which promote obesity also impair spatial memory in rodents [80], [81].

Our study terminated when the mice were 20 weeks of age (mature adulthood); however it would be of interest to ascertain whether the effects that we noted resulting from lifetime exposure to aspartame will still be apparent in an aging mouse model. Our unpublished observation, together with previous studies [81] suggests that the water maze performance of older C57Bl/6J mice decreases markedly with aging. Further studies are warranted to assess the effects of aspartame on metabolism and cognition in aging mice, and in mice from different strains. In conclusion, we have demonstrated that compared to controls, neonatal exposure of rodents to dietary aspartame, combined with chronic aspartame consumption throughout early life, may result in impairment of glucose and insulin homeostasis, together with a reduction in cognitive performance. Several gender differences were observed, with males exhibiting greater sensitivity to aspartame exposure. Our data supports previous observations that chronic exposure to aspartame may result in memory deficits in rodents.

## Supporting Information

Table S1 **Correlations between body characteristics and spatial memory variables in the MWM test.**
(PDF)
