# Supplementary material for: Gender Dimorphism in Aspartame-Induced Impairment of Spatial Cognition and Insulin Sensitivity
Source: PLoS One. 2012 Apr 3;7(4):e31570. doi: 10.1371/journal.pone.0031570 (PMC3317920; doi:10.1371/journal.pone.0031570)
Supplement: Table S1 — Correlations between body characteristics and spatial memory variables in the MWM test. (PDF) [file pone.0031570.s001.pdf]

Supplementary Table. S1

	Body Weight (6 wks)	Body Weight (17wks)	Weight Gain (%)	Fasting Insulin (uIU/ml	HOMA-IR	Fasting glucose mM	T-CHOL (mg/dL)	TG (mg/dL)	HDL-C (mg/dL)	LDL (mg/dL)	Visceral Fat (g)	Latency (s)					Thigmotaxis time (%)					Floating Time (%)					Time in Target Quadrants (%)					Platform Crosses					Annulus Crossing Index					Mean Distance to goal (m)				
												Day 1	Day 2	Day 3	Day 4	Probe Test	Day 1	Day 2	Day 3	Day 4	Probe Test	Day 1	Day 2	Day 3	Day 4	Probe Test	Day 1	Day 2	Day 3	Day 4	Probe Test	Day 1	Day 2	Day 3	Day 4	Probe Test	Day 1	Day 2	Day 3	Day 4	Probe Test	Day 1	Day 2	Day 3	Day 4	Probe Test
Body Weight (6 weeks)	1	0.888***	0.172	0.584***	0.638***	0.203	-0.168	-0.015	0.625***	-0.649***	0.372**	0.136	-0.136	-0.042	0.003	-0.025	0.024	-0.018	0.144	0.272	0.270	0.159	-0.006	0.033	-0.017	0.095	0.179	-0.137	-0.122	-0.249	0.229	-0.083	0.184	0.075	-0.180	0.000	-0.235	0.115	-0.036	-0.171	0.085	-0.083	0.084	0.124	0.293*	-0.139
Body Weight (17 weeks	0.888***	1	0.605***	0.654***	0.796***	0.370**	-0.093	-0.109	0.612***	-0.590***	0.446**	0.132	-0.092	0.075	0.029	0.076	0.137	0.113	0.263	0.291*	0.211	0.046	-0.052	0.062	0.054	0.188	0.075	-0.224	-0.162	-0.243	0.160	-0.109	0.130	0.002	-0.193	-0.120	-0.192	0.067	-0.118	-0.199	-0.027	0.007	0.217	0.218	0.345**	-0.067
Weight Gain (%)	0.172	0.605***	1	0.371**	0.584***	0.440**	0.104	-0.216	0.236	-0.144	0.322*	0.056	0.027	0.232	0.063	0.211	0.256	0.290*	0.327*	0.163	-0.010	-0.157	-0.103	0.086	0.162	0.258	-0.164	-0.245	-0.137	-0.085	-0.054	-0.092	-0.028	-0.122	-0.100	-0.265	-0.009	-0.042	-0.188	-0.133	-0.213	0.172	0.324*	0.268	0.234	0.106
Fasting Insulin (uIU/ml)	0.584***	0.654***	0.371**	1	0.821***	-0.095	-0.300*	-0.021	0.274	-0.336*	0.042	0.021	0.095	0.023	-0.076	0.017	0.106	0.085	0.002	0.046	0.001	-0.115	-0.134	-0.224	-0.128	-0.077	0.136	-0.361*	-0.135	-0.193	-0.057	-0.070	-0.020	0.094	-0.187	0.023	-0.133	-0.035	-0.051	-0.173	-0.004	-0.014	0.301*	0.080	0.135	-0.002
HOMA-IR	0.638***	0.796***	0.584***	0.821***	1	0.468**	-0.157	-0.100	0.363*	-0.365*	0.288*	0.064	0.132	0.148	0.010	0.150	0.157	0.195	0.255	0.240	0.133	-0.089	0.005	0.006	0.076	0.177	-0.024	-0.355*	-0.121	-0.329*	-0.055	-0.160	-0.034	-0.051	-0.136	-0.080	-0.212	-0.083	-0.131	-0.125	-0.052	0.069	0.333*	0.207	0.378**	0.079
Fasting glucose mM	0.203	0.370**	0.440**	-0.095	0.468***	1	0.139	-0.209	0.121	-0.024	0.403**	0.033	0.050	0.246	0.124	0.207	0.111	0.187	0.409**	0.396**	0.259	-0.045	0.174	0.368**	0.349*	0.433**	-0.234	-0.078	-0.021	-0.302*	-0.002	-0.137	-0.010	-0.260	0.053	-0.157	-0.165	-0.106	-0.182	0.060	-0.074	0.122	0.108	0.225	0.462**	0.136
T-CHOL (mg/dL)	-0.168	-0.093	0.104	-0.300*	-0.157	0.139	1	-0.021	0.233	0.025	-0.058	0.117	0.242	0.191	0.195	-0.116	-0.014	0.097	0.206	0.048	0.061	0.096	0.111	0.278*	0.214	0.202	-0.164	-0.188	-0.056	0.167	-0.114	-0.115	-0.387**	-0.160	-0.163	-0.005	-0.004	-0.201	-0.030	-0.161	0.048	0.123	0.182	0.181	-0.013	0.145
TG (mg/dL)	-0.015	-0.109	-0.216	-0.021	-0.100	-0.209	-0.021	1	0.020	-0.311*	-0.131	-0.130	0.066	-0.069	-0.077	-0.206	0.018	-0.011	-0.022	-0.210	-0.028	-0.015	-0.015	-0.161	-0.240	-0.125	0.122	-0.116	-0.030	-0.105	0.003	0.162	-0.026	0.104	-0.013	0.005	0.214	-0.103	0.083	-0.011	0.015	-0.137	0.119	-0.007	-0.059	-0.040
HDL-C (mg/dL)	0.625***	0.612***	0.236	0.274	0.363*	0.121	0.233	0.020	1	-0.926***	-0.003	0.189	-0.054	-0.065	-0.070	-0.257	-0.080	0.053	0.169	0.094	0.123	0.088	-0.074	-0.045	-0.035	0.012	0.158	-0.102	0.037	0.024	0.347*	-0.113	0.034	0.079	-0.192	0.223	-0.205	-0.018	0.042	-0.118	0.317*	-0.106	0.091	0.036	0.036	-0.252
LDL (mg/dL)	-0.649***	-0.590***	-0.144	-0.336*	-0.365*	-0.024	0.025	-0.311*	-0.926***	1	0.026	-0.118	0.094	0.130	0.139	0.281*	0.070	-0.025	-0.108	-0.020	-0.097	-0.058	0.104	0.158	0.156	0.074	-0.230	0.086	-0.041	0.048	-0.368*	0.036	-0.122	-0.146	0.151	-0.221	0.139	-0.003	-0.073	0.079	-0.303*	0.173	-0.078	0.012	-0.022	0.294*
Visceral Fat (g)	0.372**	0.446**	0.322*	0.042	0.288*	0.403**	-0.058	-0.131	-0.003	0.026	1	0.122	0.005	0.357*	0.428**	0.431**	0.129	0.265	0.48***	0.553***	0.356*	0.402**	0.340*	0.487***	0.441**	0.457**	-0.284*	-0.188	-0.349*	-0.294*	-0.134	-0.088	0.071	-0.369*	-0.244	-0.401**	-0.103	0.137	-0.384**	-0.275*	-0.359*	0.216	0.229	0.449**	0.521***	0.320*
Latency (s)																																														
Day 1	0.136	0.132	0.056	0.021	0.064	0.033	0.117	-0.130	0.189	-0.118	0.122	1	0.398**	0.389**	0.218	0.066	0.540***	0.451**	0.485***	0.401**	0.404**	0.271	0.390**	0.192	0.303*	0.332*	-0.479***	-0.256	-0.412**	-0.105	-0.252	-0.882***	-0.274	-0.341*	-0.278*	-0.062	-0.800***	-0.092	-0.352*	-0.216	0.019	0.617***	0.453**	0.574***	0.344*	0.380**
Day 2	-0.136	-0.092	0.027	0.095	0.132	0.050	0.242	0.066	-0.054	0.094	0.005	0.398**	1	0.313*	0.332*	0.131	0.374**	0.568***	0.254	0.256	0.257	0.058	0.521***	0.344*	0.363*	0.313*	-0.433**	-0.449**	-0.205	-0.122	-0.328*	-0.393**	-0.720***	-0.334*	-0.247	-0.108	-0.288*	-0.698***	-0.217	-0.218	-0.104	0.460**	0.599***	0.313*	0.222	0.423**
Day 3	-0.042	0.075	0.232	0.023	0.148	0.246	0.191	-0.069	-0.065	0.130	0.357*	0.388**	0.313*	1	0.363*	0.320*	0.325*	0.451**	0.679***	0.558***	0.481***	0.115	0.308*	0.582***	0.566***	0.507***	-0.424**	-0.606***	-0.600***	-0.274	-0.441*	-0.375**	-0.348*	-0.880***	-0.181	-0.363*	-0.332*	-0.137	-0.809***	-0.060	-0.317*	0.443**	0.629***	0.739***	0.511***	0.570***
Day 4	0.003	0.029	0.063	-0.076	0.010	0.124	0.195	-0.077	-0.070	0.139	0.428**	0.218	0.332*	0.363*	1	0.305*	0.095	0.328*	0.282*	0.522***	0.339*	0.370**	0.376**	0.446**	0.657***	0.505***	-0.403**	-0.348*	-0.539***	-0.458**	-0.563***	-0.189	-0.087	-0.330*	-0.500***	-0.419**	-0.131	-0.073	-0.276*	-0.577***	-0.354*	0.341*	0.378**	0.435**	0.587***	0.667***
Probe Test	-0.025	0.076	0.211	0.017	0.150	0.207	-0.116	-0.206	-0.257	0.281*	0.431**	0.066	0.131	0.320*	0.305*	1	0.077	0.126	0.277*	0.408**	0.163	0.205	0.347*	0.428**	0.512***	0.430**	-0.195	-0.016	-0.272	-0.201	-0.178	-0.124	0.126	-0.178	0.103	-0.769***	-0.082	0.187	-0.279*	0.136	-0.719***	0.115	-0.001	0.255	0.359*	0.266
Thigmotaxis time (%)																																														
Day 1	0.024	0.137	0.256	0.106	0.157	0.111	-0.014	0.018	-0.080	0.070	0.129	0.540***	0.374**	0.325*	0.095	0.077	1	0.525***	0.257	0.150	0.065	-0.283*	0.164	0.116	0.082	0.213	-0.563***	-0.176	-0.327*	-0.098	-0.292*	-0.499***	-0.269	-0.186	0.052	-0.139	-0.306*	-0.233	-0.290*	-0.065	-0.146	0.794***	0.389**	0.398**	0.212	0.296*
Day 2	-0.018	0.113	0.290*	0.085	0.195	0.187	0.097	-0.011	0.053	-0.025	0.265	0.451**	0.568***	0.451**	0.328*	0.126	0.525***	1	0.683***	0.572***	0.497***	0.228	0.394**	0.380**	0.456**	0.541***	-0.480**	-0.460**	-0.393**	-0.116	-0.327*	-0.384**	-0.362*	-0.382**	-0.218	-0.233	-0.214	-0.288*	-0.290*	-0.194	-0.179	0.550***	0.774***	0.687***	0.376**	0.536***
Day 3	0.144	0.263	0.327*	0.002	0.255	0.409**	0.206	-0.022	0.169	-0.108	0.488***	0.485***	0.254	0.679***	0.282*	0.277*	0.257	0.683***	1	0.739***	0.692***	0.399**	0.459**	0.517***	0.514***	0.565***	-0.373**	-0.445**	-0.437**	-0.270	-0.263	-0.453**	-0.164	-0.598***	-0.138	-0.358*	-0.424**	0.004	-0.503***	-0.029	-0.254	0.351*	0.639***	0.834***	0.611***	0.492***
Day 4	0.272	0.291*	0.163	0.046	0.240	0.396**	0.048	-0.210	0.094	-0.020	0.553***	0.401**	0.256	0.558***	0.522***	0.408**	0.150	0.572***	0.739***	1	0.806***	0.436**	0.500***	0.570***	0.666***	0.659***	-0.350*	-0.374**	-0.453**	-0.366*	-0.255	-0.369**	0.004	-0.540***	-0.303*	-0.480***	-0.389**	0.082	-0.489***	-0.228	-0.363*	0.315*	0.478**	0.669***	0.712***	0.553***
Probe Test	0.270	0.211	-0.010	0.001	0.133	0.259	0.061	-0.028	0.123	-0.097	0.356*	0.404**	0.257	0.481***	0.339*	0.163	0.065	0.497***	0.692***	0.806***	1	**0.468	0.498***	0.515***	0.486***	0.563***	-0.239	-0.355*	-0.371*	-0.265	-0.002	-0.301*	-0.096	-0.495***	-0.285*	-0.262	-0.310*	0.067	-0.328*	-0.187	-0.106	0.227	0.473***	0.589***	0.580***	0.369**
Floating Time (%)																																														
Day 1	0.159	0.046	-0.157	-0.115	-0.089	-0.045	0.096	-0.015	0.088	-0.058	0.402**	0.271	0.058	0.115	0.370**	0.205	-0.283*	0.228	0.399**	0.436**	0.468***	1	0.508***	0.427**	0.471***	0.425**	-0.111	-0.149	-0.214	-0.035	-0.123	-0.222	0.072	-0.077	-0.342*	-0.259	-0.181	0.243	0.061	-0.225	-0.191	-0.025	0.188	0.319*	0.245	0.342*
Day 2	-0.006	-0.052	-0.103	-0.134	0.005	0.174	0.111	-0.015	-0.074	0.104	0.340*	0.390**	0.521***	0.308*	0.376**	0.347*	0.164	0.394**	0.459**	0.500***	0.498***	0.508***	1	0.602***	0.566***	0.619***	-0.402**	-0.221	-0.185	-0.200	-0.190	-0.360*	-0.278*	-0.282*	-0.190	-0.288*	-0.297*	-0.058	-0.114	-0.130	-0.243	0.356*	0.316*	0.334*	0.420**	0.433**
Day 3	0.033	0.062	0.086	-0.224	0.006	0.369**	0.278*	-0.161	-0.045	0.158	0.487***	0.192	0.344*	0.582***	0.446**	0.428**	0.116	0.380**	0.517***	0.570***	0.515***	0.427**	0.602***	1	0.780***	0.771***	-0.390**	-0.287*	-0.336*	-0.183	-0.219	-0.161	-0.314*	-0.535***	-0.174	-0.421**	-0.128	-0.133	-0.255	-0.078	-0.332*	0.314*	0.337*	0.471***	0.464***	0.490***
Day 4	-0.017	0.054	0.162	-0.128	0.076	0.349*	0.214	-0.240	-0.035	0.156	0.441**	0.303*	0.363*	0.566***	0.657***	0.512***	0.082	0.456**	0.514***	0.666***	0.486***	0.471***	0.566***	0.780***	1	0.843***	-0.428**	-0.402**	-0.401**	-0.284*	-0.442**	-0.324*	-0.160	-0.498***	-0.359*	-0.421**	-0.296*	-0.043	-0.326*	-0.264	-0.356*	0.341*	0.440**	0.482***	0.576***	0.689***
Probe Test	0.095	0.188	0.258	-0.077	0.177	0.433**	0.202	-0.125	0.012	0.074	0.457**	0.332*	0.313*	0.507***	0.505***	0.430**	0.213	0.541***	0.565***	0.659***	0.563***	0.425**	0.619***	0.771***	0.843***	1	-0.406**	-0.338*	-0.363*	-0.186	-0.322*	-0.317*	-0.167	-0.411**	-0.264	-0.390**	-0.257	-0.015	-0.255	-0.202	-0.286*	0.372**	0.448**	0.509***	0.536***	0.641***
Time in Target Quadrants (%)																																														
Day 1	0.179	0.075	-0.164	0.136	-0.024	-0.234	-0.164	0.122	0.158	-0.230	-0.284*	-0.479***	-0.433**	-0.424**	-0.403**	-0.195	-0.563***	-0.480***	-0.373**	-0.350*	-0.239	-0.111	-0.402**	-0.390**	-0.428**	-0.406**	1	0.261	0.391**	0.289*	0.425**	0.481***	0.278*	0.382**	0.134	0.229	0.332*	0.158	0.314*	0.267	0.230	-0.897***	-0.403**	-0.495***	-0.470***	-0.516***
Day 2	-0.137	-0.224	-0.245	-0.361*	-0.355*	-0.078	-0.188	-0.116	-0.102	0.086	-0.188	-0.256	-0.448**	-0.606***	-0.348*	-0.016	-0.176	-0.460**	-0.445**	-0.374**	-0.355*	-0.149	-0.221	-0.287*	-0.402**	-0.338*	0.261	1	0.463***	0.203	0.409**	0.273	0.413**	0.464***	0.472***	0.137	0.211	0.302*	0.447**	0.400**	0.133	-0.268	-0.871***	-0.574***	-0.317*	-0.495***
Day 3	-0.122	-0.162	-0.137	-0.135	-0.121	-0.021	-0.056	-0.030	0.037	-0.041	-0.350*	-0.412**	-0.205	-0.600***	-0.539***	-0.272	-0.327*	-0.393**	-0.437**	-0.453**	-0.371**	-0.214	-0.185	-0.336*	-0.401**	-0.363*	0.391**	0.463***	1	0.437**	0.381**	0.390**	0.102	0.410**	0.227	0.429**	0.362*	0.008	0.475***	0.287*	0.383**	-0.386**	-0.524***	-0.796***	-0.574***	-0.452**
Day 4	-0.249	-0.243	-0.085	-0.193	-0.330*	-0.302*	0.167	-0.105	0.024	0.048	-0.294*	-0.105	-0.122	-0.274	-0.458**	-0.201	-0.098	-0.116	-0.270	-0.366*	-0.265	-0.035	-0.200	-0.183	-0.284*	-0.186	0.289*	0.203	0.437**	1	0.366*	0.209	-0.098	0.274	0.013	0.342*	0.243	0.007	0.270	0.133	0.337*	-0.206	-0.193	-0.372**	-0.846***	-0.369**
Probe Test	0.229	0.160	-0.054	-0.057	-0.055	-0.002	-0.114	0.003	0.347*	-0.368*	-0.134	-0.252	-0.328*	-0.441**	-0.563***	-0.178	-0.292*	-0.327*	-0.263	-0.255	-0.002	-0.123	-0.190	-0.219	-0.442**	-0.322*	0.425**	0.409**	0.381**	0.366*	1	0.369**	0.265	0.372**	0.233	0.358*	0.254	0.202	0.327*	0.314*	0.460**	-0.430**	-0.446**	-0.424**	-0.397**	-0.891***
Platform Crosses																																														
Day 1	-0.083	-0.109	-0.092	-0.070	-0.160	-0.137	-0.115	0.162	-0.113	0.036	-0.088	-0.882***	-0.393**	-0.375***	-0.189	-0.124	-0.499***	-0.384**	-0.453**	-0.369**	-0.301*	-0.222	-0.360*	-0.161	-0.323*	-0.317*	0.481***	0.273	0.390**	0.209	0.369**	1	0.179	0.253	0.056	0.091	0.878***	0.042	0.338*	0.046	0.060	-0.550***	-0.402**	-0.521***	-0.389**	-0.434**
Day 2	0.184	0.130	-0.028	-0.020	-0.034	-0.010	-0.387**	-0.026	0.034	-0.122	0.071	-0.274	-0.720***	-0.348*	-0.087	0.126	-0.269	-0.362*	-0.164	0.004	-0.096	0.072	-0.278*	-0.314*	-0.160	-0.167	0.278*	0.414**	0.102	-0.098	0.265	0.179	1	0.389**	0.242	-0.087	0.068	0.846***	0.161	0.205	-0.040	-0.356*	-0.496***	-0.210	0.034	-0.320*
Day 3	0.075	0.002	-0.122	0.094	-0.051	-0.260	-0.160	0.104	0.079	-0.146	-0.369*	-0.341*	-0.334*	-0.879***	-0.330*	-0.178	-0.186	-0.382**	-0.598***	-0.540***	-0.495***	-0.077	-0.282*	-0.535***	-0.498***	-0.411**	0.382**	0.464***	0.410**	0.274	0.372**	0.253	0.389	1	0.290*	0.193	0.297*	0.236	0.804***	0.137	0.152	-0.399**	-0.539***	-0.592***	-0.483***	-0.515***
Day 4	-0.180	-0.193	-0.100	-0.187	-0.136	0.053	-0.163	-0.013	-0.192	0.151	-0.244	-0.278*	-0.247	-0.181	-0.500***	0.103	0.052	-0.218	-0.138	-0.303*	-0.285*	-0.342*	-0.190	-0.174	-0.359*	-0.264	0.134	0.472***	0.227	0.013	0.233	0.056	0.242	0.290*	1	-0.030	0.081	0.183	0.137	0.879***	-0.066	-0.150	-0.451**	-0.200	-0.161	-0.365*
Probe Test	0.000	-0.120	-0.265	0.023	-0.080	-0.157	-0.005	0.005	0.223	-0.221	-0.401**	-0.062	-0.108	-0.363*	-0.419**	-0.769***	-0.139	-0.233	-0.358*	-0.479**	-0.262	-0.259	-0.288*	-0.421**	-0.421**	-0.389**	0.229	0.137	0.429**	0.342*	0.358*	0.091	-0.087	0.193	-0.030	1	-0.030	-0.167	0.235	0.032	0.953***	-0.146	-0.142	-0.434**	-0.487***	-0.433*
Annulus Crossing Index																																														
Day 1	-0.235	-0.192	-0.009	-0.133	-0.212	-0.165	-0.004	0.214	-0.205	0.139	-0.103	-0.800***	-0.288*	-0.332*	-0.131	-0.082	-0.306*	-0.214	-0.424**	-0.389**	-0.310*	-0.181	-0.297*	-0.128	-0.296*	-0.257	0.332*	0.211	0.362*	0.243	0.254	0.878***	0.068	0.297*	0.081	-0.030	1	0.007	0.405**	0.019	-0.060	-0.384**	-0.304*	-0.473***	-0.420**	-0.311*
Day 2	0.115	0.067	-0.042	-0.035	-0.083	-0.106	-0.201	-0.103	-0.018	-0.003	0.137	-0.092	-0.698***	-0.137	-0.073	0.187	-0.233	-0.288*	0.004	0.082	0.067	0.243	-0.058	-0.133	-0.043	-0.015	0.158	0.302*	0.008	0.007	0.202	0.042	0.846***	0.236	0.183	-0.167	0.007	1	0.085	0.187	-0.110	-0.204	-0.385**	-0.038	0.065	-0.188
Day 3	-0.036	-0.118	-0.188	-0.051	-0.131	-0.182	-0.030	0.083	0.042	-0.073	-0.384**	-0.352*	-0.217	-0.809***	-0.276*	-0.279*	-0.290*	-0.290*	-0.503***	-0.489***	-0.328*	0.061	-0.114	-0.255	-0.327*	-0.255	0.314*	0.447**	0.475***	0.270	0.327*	0.338*	0.161	0.804***	0.137	0.235	0.405**	0.085	1	0.018	0.222	-0.354*	-0.455**	-0.575***	-0.416**	-0.401**
Day 4	-0.171	-0.199	-0.133	-0.173	-0.125	0.060	-0.161	-0.011	-0.118	0.079	-0.275*	-0.216	-0.218	-0.060	-0.577***	0.136	-0.065	-0.194	-0.029	-0.228	-0.187	-0.225	-0.130	-0.078	-0.264	-0.202	0.267	0.400**	0.287*	0.133	0.314*	0.046	0.205	0.137	0.879***	0.032	0.019	0.187	0.018	1	0.017	-0.285*	-0.399**	-0.204	-0.232	-0.391**
Probe Test	0.085	-0.027	-0.213	-0.004	-0.052	-0.074	0.048	0.015	0.317*	-0.303*	-0.359*	0.019	-0.104	-0.317*	-0.354*	-0.719***	-0.146	-0.179	-0.254	-0.363*	-0.106	-0.191	-0.243	-0.332*	-0.356*	-0.286*	0.230	0.133	0.383**	0.337*	0.460**	0.060	-0.040	0.152	-0.066	0.953***	-0.060	-0.110	0.222	0.017	1	-0.140	-0.096	-0.348*	-0.410*	-0.467**
Mean Distance to goal (m)																																														
Day 1	-0.083	0.007	0.172	-0.014	0.069	0.122	0.123	-0.137	-0.106	0.173	0.216	0.617***	0.460**	0.443**	0.341*	0.115	0.794***	0.550***	0.351*	0.315*	0.227	-0.025	0.356*	0.314*	0.341*	0.372**	-0.897***	-0.268	-0.386**	-0.206	-0.430**	-0.550***	-0.355*	-0.399**	-0.150	-0.146	-0.384**	-0.204	-0.354*	-0.285*	-0.140	1	0.471**	0.506***	0.398**	0.505***
Day 2	0.084	0.217	0.323*	0.301*	0.333*	0.108	0.182	0.119	0.091	-0.078	0.229	0.453**	0.599***	0.629***	0.378**	-0.001	0.389**	0.774**	0.639***	0.478***	0.473***	0.188	0.316*	0.337*	0.440**	0.448**	-0.403**	-0.871***	-0.524***	-0.193	-0.446***	-0.402**	-0.496***	-0.539***	-0.451**	-0.142	-0.304*	-0.385**	-0.455**	-0.399**	-0.096	0.471***	1	0.747***	0.401**	0.591***
Day 3	0.124	0.218	0.268	0.080	0.207	0.225	0.181	-0.007	0.036	0.012	0.449**	0.574***	0.313*	0.739***	0.435**	0.255	0.398**	0.687***	0.834***	0.669***	0.589***	0.319*	0.334*	0.471***	0.482**	0.509***	-0.495***	-0.574***	-0.796***	-0.372**	-0.424**	-0.521***	-0.210	-0.592***	-0.200	-0.434**	-0.473***	-0.038	-0.575***	-0.204	-0.348*	0.506***	0.747***	1	0.655***	0.580***
Day 4	0.293*	0.345*	0.234	0.135	0.378**	0.462***	-0.013	-0.059	0.036	-0.022	0.521***	0.344*	0.222	0.511***	0.587***	0.359*	0.212	0.376**	0.611***	0.712***	0.580***	0.245	0.420**	0.464***	0.576***	0.536***	-0.470***	-0.317*	-0.574***	-0.846***	-0.397**	-0.389**	0.034	-0.483***	-0.161	-0.487***	-0.420**	0.065	-0.416**	-0.232	-0.410**	0.398**	0.401**	0.655***	1	0.554***
Probe Test	-0.139	-0.067	0.106	-0.002	0.079	0.136	0.145	-0.040	-0.252	0.294*	0.320*	0.380**	0.423**	0.570***	0.667***	0.266	0.296*	0.536***	0.492***	0.553***	0.369**	0.342*	0.433**	0.490***	0.689***	0.641***	-0.516***	-0.495***	-0.452**	-0.369**	-0.891***	-0.434**	-0.320*	-0.515***	-0.365*	-0.433**	-0.311*	-0.188	-0.401**	-0.391**	-0.467***	0.505***	0.591***	0.580***	0.554***	1

**. Correlation is significant at the 0.01 level (2-tailed).

*. Correlation is significant at the 0.05 level (2-tailed).
